# Microenvironmental Metabolites in the Intestine: Messengers between Health and Disease

**DOI:** 10.3390/metabo12010046

**Published:** 2022-01-07

**Authors:** Antonio Enrico Zaurito, Markus Tschurtschenthaler

**Affiliations:** 1 Institute for Experimental Cancer Therapy, Center for Translational Cancer Research (TranslaTUM), Klinikum Rechts der Isar, Technical University of Munich, 81675 Munich, Germany; antonio.zaurito@tum.de; 2 Department of Internal Medicine II, Klinikum Rechts der Isar, Technical University of Munich, 81675 Munich, Germany; 3 Division of Translational Cancer Research, German Cancer Research Center (DKFZ), German Cancer Consortium (DKTK), 69120 Heidelberg, Germany

**Keywords:** colorectal cancer, intestinal inflammation, oncometabolites, microbiota

## Abstract

The intestinal mucosa is a highly absorptive organ and simultaneously constitutes the physical barrier between the host and a complex outer ecosystem. Intestinal epithelial cells (IECs) represent a special node that receives signals from the host and the environment and translates them into corresponding responses. Specific molecular communication systems such as metabolites are known to transmit information across the intestinal boundary. The gut microbiota or food-derived metabolites are extrinsic factors that influence the homeostasis of the intestinal epithelium, while mitochondrial and host-derived cellular metabolites determine the identity, fitness, and regenerative capacity of IECs. Little is known, however, about the role of intrinsic and extrinsic metabolites of IECs in the initiation and progression of pathological processes such as inflammatory bowel disease and colorectal cancer as well as about their impact on intestinal immunity. In this review, we will highlight the most recent contributions on the modulatory effects of intestinal metabolites in gut pathophysiology, with a particular focus on metabolites in promoting intestinal inflammation or colorectal tumorigenesis. In addition, we will provide a perspective on the role of newly identified oncometabolites from the commensal and opportunistic microbiota in shaping response and resistance to antitumor therapy.

## 1. Introduction

### 1.1. The Intestinal Epithelium Functions as a Barrier and Nutrient-Absorbing Organ

The intestinal epithelium is recognized as the largest mucosal surface of the body (with roughly 400 m^2^ area), organized in a monolayer of columnar epithelial cells [1]. The intestinal epithelium is characterized by finger-shaped “crypt-villus” structures that are present over the entire length of the intestine with exceptional variations in the colon, where villi are lacking due to the reduced rate of nutrient absorption occurring in this region. Villi are defined as elongated structures that protrude into the lumen of the intestinal tract and alternate with depressions known as “crypts of Lieberkühn”. Villi can increase the surface area of the small intestine of another 600 times due to additional protrusions on intestinal epithelial cells (IECs, enterocytes) called microvilli [2]. Villi and microvilli together maximize nutrient uptake in the intestine by potentiating the available area. All vertebrates share the same villi morphogenesis: they originate from the endoderm, a flattened line of cells that gives rise to all the internal parts of organs and cavities exposed to an external environment [3]. The integrity of each individual IEC and villus and their functions are critical to achieving optimal nutrient absorption, secretion, and luminal sensing in the small intestine. The mechanical and physiological stress to which the intestine is constantly exposed makes this tissue highly insulted, so that all differentiated enterocytes are replaced every 3–4 days to ensure a good performance of the intestinal functions [4]. A pool of pluripotent intestinal stem cells (ISCs) residing in the intestinal crypt bottom is responsible for the renewal of the mucosal surface. Barker et al. identified Lgr5^+^ ISCs, which are responsible for the regeneration of all cell types that make up the intestinal epithelium [5]. Continuous cycling of ISCs gives rise to transit-amplifying cells (TACs), which are the progenitors of the absorptive and secretory cell lineages. Opposing Wnt (Wingless-related integration site) and BMP (bone morphogenetic proteins) signaling gradients along the crypt-villus axis ensure stemness and differentiation into specialized IECs, respectively. The absorptive lineage includes enterocytes, which are the most abundant cell population in the intestinal epithelium and have functions of absorbing nutrients and to secreting immunoglobins. In addition, the secretory lineages comprise enteroendocrine cells (EECs), Tuft cells, and Goblet cells [6]. Goblet cells and Paneth cells (PCs) ensure the integrity of the intestinal barrier by producing a layer of mucus as a first line defense against infiltration and translocation of microbes or the secretion of antimicrobial peptides, respectively. EECs produce peptides or hormones in response to various stimuli, connecting the central and enteric neuroendocrine system, and regulating the digestive function [7].

### 1.2. Principal Concepts of Intestinal Homeostasis and Metabolism

Due to the high renewal rate of the intestinal epithelium and its functions as a barrier and in nutrient uptake, IECs exhibit a high energy consumption [8]. The main source of energy is food, and influences in this process can rapidly affect homeostasis and metabolism of the intestinal epithelium. Moreover, the direct contact of IECs with metabolites in the gut lumen also plays a role in shaping the gut integrity and mucosa immune responses of the epithelium [9,10]. In addition to the above-mentioned BMP and Wnt signaling, various intrinsic and extrinsic metabolic changes play a fundamental role in regulation of intestinal homeostasis [11]. Particularly, caloric restriction (CR), fasting, glucose supplemented diet, and high-fat diet are examples of metabolic changes affecting the metabolic status of IECs. CR has been demonstrated to have a direct impact on ISC functions by increasing the number of stem cells by fine-tuning mTOR signaling and protecting them from DNA damage [12]. Fasting itself can also improve ISC proliferation capacity. Mihaylova and colleagues suggested that short-term fasting in both young and adult mice increased ISC functions mediated by fatty acid oxidation (FAO) [13]. Conversely, high-fat diet (HFD) was found to have different outcomes. In an HFD, activation of peroxisome proliferator-activated receptors signaling (PPARs), a family of lipid activated nuclear receptors, increased intestinal stemness [14]. However, FAO-derived metabolites create a favorable environment for the development of intestinal tumors, as demonstrated in mice with a conditional knockout of adenomatous polyposis coli (*Apc*-iKO) in Lgr5^+^ expressing ISCs when administered a HFD compared to the control condition [15]. Another study in mice also highlighted the controversial role of HFD, which on the one hand increases the number of ISCs in both the small and large intestines and on the other hand impairs the integrity of the epithelial barrier [16]. Moreover, HFD has also been linked to changes in the gut microbiota and a predisposition in the offspring to a more severe colitis induced by dextran sodium sulfate (DSS) [17]. Glucose supplemented diet is also thought to be involved in intestinal dysfunctions. Aliluev and colleagues described how a diet high in fat and sugar changed the gut mucosa and ISC niche through hyperproliferation and accelerated turnover of ISC differentiation [18]. The mitochondrial activity of ISCs is another rheostat that helps to adapt to metabolic changes. Within the ISC niche, stem cells and differentiated cells show different metabolic phenotypes. In fact, Lgr5^+^ cells exhibit a more pronounced oxidative phosphorylation (OXPHOS) activity than differentiated cells like PCs. Moreover, PCs closely interact with ISCs to maintain a balanced intestinal homeostasis through the production of lactate. Lactate, which is obtained from the glycolytic metabolism, stimulates the OXPHOS pathway in ISCs, and thus promotes differentiation processes [19]. Nevertheless, disruption of mitochondrial functions has been concomitantly linked to inflammatory bowels disease (IBD) [20]. Specifically, Khaloian et al. showed that inflammation-associated mitochondrial dysfunction caused a metabolic imbalance, which ultimately led to reduced stemness and dysfunctional PCs [21]. Taken together, these studies support the idea that intestinal homeostasis is controlled by both intrinsic (mitochondrial metabolism) and extrinsic (nutrient supply and inflammatory molecules) metabolic changes. Maladaptation of cellular metabolism to microenvironmental stimuli predisposes the way for the onset of intestinal diseases.

## 2. Metabolism and Intestinal Diseases

### 2.1. Metabolites Affecting Intestinal Barrier Functions

The intestinal epithelium constitutes the barrier to the luminal environment of the gut. Ensuring the integrity of the intestinal barrier is critical to fulfill all needs, including maintaining good permeability and blocking the entry of pathogens [22]. Several factors can affect this interface, including host and luminal gut-microbiota-derived metabolites [23]. The intestinal barrier must adapt to a plethora of changes in order to maintain a homeostatic status. Therefore, several mechanisms are present to challenge any ongoing metabolic changes. High energy consumption and in particular fluctuation in O_2_ level are key indicators of a high performing intestinal barrier. Of interesting note is the O_2_ gradient present along the crypt-villus axis and along the intestinal tract. For example, the small intestine is characterized by high oxygen perfusion mediated by villi and microvilli. In sharp contrast, the large intestine is almost anaerobic. That is why we refer to the gut as a site of “physiological hypoxia” [24]. However, alterations in the oxygen metabolism during inflammatory conditions (i.e., IBD) can comprise the integrity and permeability of the intestinal barrier. In this regard, the role of Hypoxia inducible factor (HIF) tends to have a protective role in chronic inflammation [25]. It has been demonstrated that HIF triggers pathways involved in mucus secretion and barrier fortification [26,27]. Food-derived metabolites have also been implicated in shaping the structure and functions of the intestinal barrier. Dietary fiber are carbohydrate polymers that cannot be hydrolyzed by endogenous enzymes in the intestine. However, the microbiota that populates the luminal side of the intestine can use them as substrates for specific enzymatic processes such as fermentation. It has been demonstrated in vivo that the availability of dietary fiber can counteract the consumption of glycans (principal component of the mucus layer) by bacteria, as the gut microbiota can use glycans as an energy source and therefore break down the mucus layer, which in turn increases the intestinal permeability [28,29]. Soluble fibers can eventually be fermented by the luminal microbiota to produce short-chain fatty acids (SCFAs) such as propionate, butyrate, and acetate, which are metabolites involved in gut-immune homeostasis. Their function is to maintain the status quo of the intestinal barrier and to control the differentiation and proliferation of T regulatory cells (Tregs). Tregs are a subset of CD4^+^ T helper cells that help the body to keep control during inflammation-driven T helper activities. Therefore, a lack of SCFAs results in reduced presence of Tregs in the tissue that indirectly favor the induction and proliferation of the Th17 cells, thus promoting inflammatory events in the intestinal mucosa [30,31]. Additionally, a recent work demonstrated a mechanism by which microbiota-derived butyrate maintains intestinal homeostasis [32]. Specifically, butyrate downregulates hexokinase-2 (HK2), which is upregulated in inflammation, via histone deacetylase 8 (HDAC8), thus alleviating colitis [32]. Vitamins such as vitamin A and D have also been implicated in the modulation of mucosal barrier integrity by regulating epithelial tight junctions and bacteria load. Administration of vitamin A to IPEC-J2 cells as part of lipopolysaccharide (LPS) treatment was found to improve transepithelial electrical resistance (TEER), attenuate LPS activity on the intestinal barrier and promote the expression of adhesion molecules [33]. Vitamin D has also been studied in the context of intestinal disease such as ulcerative colitis (UC). Stio et al. demonstrated that vitamin D can reduce the cytokines levels and upregulate claudine-2, 4, and 7, which are important for intestinal barrier integrity [34]. Finally, a study in mice suggested that either a lack of vitamin D or the inability of vitamin D to bind its receptor resulted in loss of barrier integrity and increased inflammation through modulation of the microbiota [35]. HFD is another factor that increases the permeability of the intestinal barrier by modulating tight junctions and microbiota. Rats fed a high fat, high-calorie diet were prone to chronic systemic inflammation. In addition, upregulation of Toll like receptor 4 (TLR4) and increased levels of LPS were observed along with ileal inflammation [36]. Altogether, these data underline the critical role of extrinsic metabolites derived from food or the luminal microbiota in influencing intestinal barrier functions.

### 2.2. Metabolism and Colorectal Cancer Tumorigenesis

**Figure 1:**
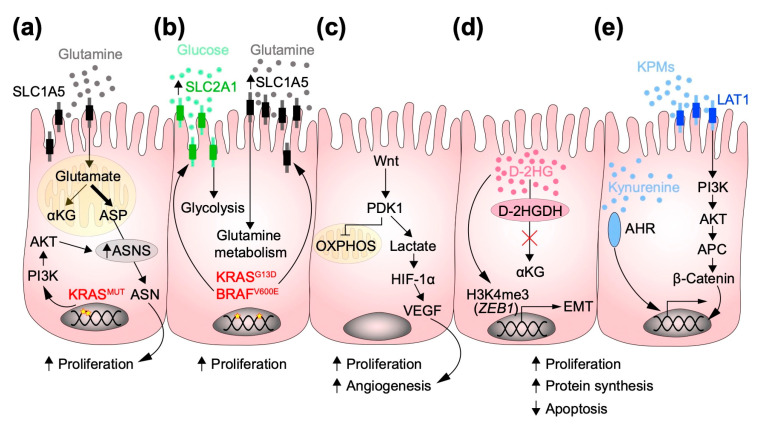
Schematic representation of how autonomous oncometabolites affect the development of CRC: (**a**) In the absence of glucose, the oncogenic *Kras* signaling upregulates the expression of asparagine synthetase (ASNS), the enzyme involved in conversion of aspartate (ASP) to asparagine (ASN), which in turn promotes proliferation. (**b**) KRAS and BRAF mutations lead to upregulation of the glucose and glutamine transporters SLC2A1 and SLC1A5 (Solute carrier family 2 and 5 member), respectively, which leads to an increased glutamine and glucose uptake that is crucial to sustain cancer cell proliferation. (**c**) Activation of the Wnt pathway suppresses oxidative phosphorylation (OXPHOS) and favors activation of the anaerobic glycolytic pathway, which leads to increased expression of vascular endothelial growth factor (VEGF), which in turn supports tumor angiogenesis and proliferation. (**d**) The accumulation of D-2-hydroxyglutarate (D-2HG) stimulates the expression of EMT (epithelial-mesenchymal transition) markers and anti-apoptotic genes, thereby promoting proliferation, invasiveness, and reduced apoptosis. (**e**) Kynurenine pathway metabolites (KPMs) accumulate in the cell due to the overexpression of the membrane solute carrier LAT1 (L-type/large amino acid transporter 1), which trigger the PI3K-AKT and β-catenin pathways to activate genes involved in proliferation. Moreover, kynurenine promotes the nuclear translocation of AHR (Aryl hydrocarbon receptor), which process leads to increased proliferation.

Besides improvements in cancer therapies like immune checkpoint blockade (ICB), radiotherapy, chemotherapy, and preventive screening tests, colorectal cancer (CRC) is still one of the leading causes of cancer death worldwide [37]. Development of CRC is influenced by several genetic, epigenetic, and environmental as well as diet-related factors [38]. Sporadic CRC development is a multi-step process in which inactivation of tumor suppressor genes or activation of oncogenes leads to cellular transformation and malignancy. Early inactivation of APC, the most common mutated gene in CRC, promotes the formation of adenomas and the subsequent acquisition of mutations in key oncogenic drivers such as *KRAS*, *TP53,* and *SMAD4* leads to progression to invasive adenocarcinomas [39,40]. Recently, a better transcriptomic analysis has improved our knowledge about CRC and the crosstalk with the tumor microenvironment. CRC has thereby been stratified into four consensus molecular subtypes (CMS), namely, (i) an immune subtype (CMS1; hypermutated, microsatellite instable (MSI), strong immune activation), (ii) a canonical subtype (CMS2; epithelial, marked Wnt and MYC signaling activation), (iii) a metabolic subtype (CMS3; epithelial, metabolic dysregulation), and (iv) a mesenchymal subtype (CMS4; TGF-β activation, stromal invasion and angiogenesis) [41]. Of note is the introduction of a metabolic subtype, which underscores the fact that metabolic rewiring in cancer constitutes a hallmark of CRC. Altered or deregulated metabolic pathways have recently been linked with tumorigenesis, which provide cancer cells in particular with some advantages (e.g., energy sources) in order to displace normal cells in their proliferation [42]. In the next paragraphs, we aim to highlight the role of metabolism and “metabolic messengers” such as oncometabolites as molecular drivers of CRC development (Figure 1).

### 2.3. Oncogenic Signaling and Metabolic Pathways Involved in CRC Tumorigenesis

The concept that the multiple acquisitions of genetic alterations, such as mutations in oncogenes and tumor suppressor genes, reprograms the metabolism of cancer cells has long been known. Already in 1997, Shim et al. deciphered the mechanism of aerobic glycolysis of tumors. In their work, they suggested that Lactate dehydrogenase A (LDH-A), an enzyme involved in converting lactate to pyruvate in normal cells under normoxic conditions, cooperates with c-Myc to promote the acquisition of neoplastic traits [43]. This work then paved the way for many other studies that identified metabolic genes that are involved in the maintenance of tumor cells. Hence, metabolic reprogramming in cancer is now widely accepted as a new paradigm for cancer development [44,45,46]. Several metabolic pathways are altered in CRC, including the tricarboxylic acid cycle (TCA), amino-acid turnover, glycolysis, and gluconeogenesis, but also changes within the lipid and steroid metabolism have been described [47]. A relationship between oncogenic signaling and reprogramming of carbon metabolism has already been shown in several cancer types. For example, KRAS-driven pancreatic ductal adenocarcinoma (PDAC) showed a profound modulation of enzymes involved in redirecting glucose metabolism to anabolic pathways, thereby promoting PDAC tumorigenesis [48]. In CRC, KRAS, and BRAF signaling pathways have been characterized as potent drivers of metabolic rewiring. KRAS mutation in human CRC cells can privilege the amino-acid consumption from aspartate to asparagine and upregulate asparagine synthetase (ASNS), the enzyme involved in the asparagine biosynthesis (Figure 1a) [49]. Experiments with human specimens and CRC cell lines showed that *KRAS* and *BRAF* mutations are implicated in glucose and lactate metabolism. Specifically, KRAS led to the upregulation of amino-acid solute carrier like SLC2A1 (Solute carrier family 2 member 1) and SLC1A5 (Solute carrier family 2 member 5 or ASCT2), a family of transporters involved in cellular growth and proliferation (Figure 1b) [50]. The Wnt signaling pathway has also been shown to play a central role in cancer metabolism. Pate and colleagues identified a mechanism by which Wnt maintains cancer cell proliferation. Wnt was found to interact with its downstream target pyruvate dehydrogenase kinase 1 (PDK1), which blocks OXPHOS to promote glycolysis. The lactate produced by this reaction then drives angiogenesis via the HIF-1α-VEGF (Vascular endothelial growth factor) pathway and thus supports tumor proliferation (Figure 1c) [51]. In the Wnt signaling pathway, *APC* mutations have also been suggested to influence the metabolic profile of CRC. Using a zebrafish model of colon cancer, Sandoval and colleagues demonstrated that pyruvate metabolism is modulated in APC-driven tumor development. Mitochondrial pyruvate carrier 1 and 2 (MCP1/2), which are important factors in intestinal differentiation, were found to be downregulated in the *apc*^mcr^ zebrafish model. Rescue of MCP1 was enough to restore intestinal differentiation [52]. Taken together, these observations corroborate the paradigm in which various genetic alterations drive tumor development while modulating the cell’s metabolism.

### 2.4. Oncometabolites: Metabolic Determinants of CRC Initiation and Progression

Over the past decade, significant advances have been made in improving techniques that enable metabolites to be identified and quantified in biological samples [53]. Metabolomics experiments, along with the identification of mutations in mitochondrial genes that are fundamental to cell metabolism, have led to the identification of specific metabolites that accumulate specifically in cancer cells compared to non-malignant cells [54,55,56]. Metabolites are intermediate products of various metabolic pathways and mostly the building blocks for bioenergetics reactions and the biosynthesis of macromolecules. Nevertheless, high concentrations of these metabolites can potentially lead to cell transformation, and thereby cause cancer [57]. In this case, the metabolites are referred to as “oncometabolites”. Studying the direct relationship between oncometabolites and cancer is a relatively new area of research in cancer biology. However, multiple pieces of evidence suggest that metabolic changes, accumulation of oncometabolites and cancer development are linked [58,59]. The aim of the next paragraphs is to provide an overview of any major scientific contribution where compelling evidence exists between the accumulation of oncometabolites and the mutation in a particular cancer gene or the development of CRC.

### 2.5. 2-Hydroxyglutarate (2-HG) and Kynurenine (Kyn): Oncometabolites in CRC

2-hydroxyglutarate (2-HG) is an important factor in the TCA because it competes with α-ketoglutarate (α-KG) and thus inhibits the production of isocitrate, so that citrate is lacking for bioenergetic processes. This conversion is catalyzed by two specific enzymes, isocitrate dehydrogenase 1 and 2 (IDH-1/2). Mutations occurring in genes encoding for IDH-1/2 lead to the production of 2-HG [60]. Evidence that mutations in *IDH* genes were frequently found in cancer led to the suggestion that 2-HG may be involved in cancer development. Due to the presence of a chiral carbon in position 2, two enantiomers have been identified, L-2HG and D-2HG. Aside from being generated by the reduction in a ketone group of α-KG to a hydroxyl group, other enzymes are also involved in 2-HG metabolism. Lactate dehydrogenase A (LDH-A) and malate dehydrogenase 1 (MDH-1) in the cytoplasm can process α-KG into L-2HG. In mitochondria, α-KG is converted into L-2HG by malate dehydrogenase 2 (MDH-2). It is important to emphasize that 2-HG is also present under physiological conditions [61]. However, it has been shown that the accumulation of this metabolite promotes DNA hypermethylation and thereby fuels development of clear cell renal cell carcinoma (ccRCC), glioma, and leukemia [62,63,64]. Apart from its pro-tumorigenic effect, 2-HG has also been reported to be able to manipulate adaptive and innate immunity while creating an immunosuppressive microenvironment [65,66]. Brain tumors often show mutations in *IDH* genes and thus exhibit a high conversion of α-KG into the oncometabolite form D-2HG [59]. The presence of these mutations in glioma tumors have been associated with a reduced infiltration of CD8^+^ T cells within the core of the tumor compared with tumors with functional IDH-1/2. This can be explained by the downregulation of CD8^+^-associated genes and T cell-mediated chemokines [67]. Moreover, *IDH* mutations favor Treg accumulation while interfering with Th17 polarization by shifting bioenergetics processes (OXPHOS instead of glycolysis) and HIF-1α destabilization [68]. Finally, D-2HG inhibited classical and alternative pathways of complement activation [69].

The role of 2-HG has also been explored in CRC. Colvin et al. demonstrated that the oncometabolite D-2HG, but not its enantiomer L-2HG, plays a role in CRC progression and metastatic processes. In CRC cell lines, they found an accumulation of D-2HG. In addition, the administration of an octylester derivate of D-2HG to the HCT116 cell line, which does not harbor any mutations in the *IDH* genes, changed the shape of the cells to a more mesenchymal phenotype and thus favored epithelial-to-mesenchymal transition (EMT). In addition, they found that D-2HG is able to epigenetically modify the promoter of *ZEB1* (Zinc finger E-box-binding homeobox 1) by DNA trimethylation. Finally, they validated this results in patients and showed that the likelihood of developing metastases is more common when the concentration of D-2HG in the tumor is higher (Figure 1d) [70]. One of the causes for the occurrence of CRC is a preceding chronic intestinal inflammation. In fact, patients with IBD such as UC or Crohn’s disease (CD) have a higher lifetime risk of developing CRC [71]. Interestingly, enzymes involved in the TCA, such as IDH-1, have been found to be mutated in IBD-associated adenocarcinomas, raising the question of whether inflammation and oncometabolites may be linked to drive carcinogenesis. Using colitis-associated colon cancer (CAC) mouse models, Han et al. demonstrated that elevated D-2HG levels in urine specimens correlated with a higher tumor propensity in the intestine. Mechanistically, D-2-hydroxyglutarate dehydrogenase (D2HGDH), the enzymes involved in the elimination of D-2HG, had a reduced expression in biopsies from UC patients and thus the D-2HG metabolite levels were increased [72].

The kynurenine (Kyn) pathway is well known as it is responsible for almost all the tryptophan metabolism. Tryptophan is an essential amino acid and must therefore be ingested from diet. The Kyn pathway comprises three major enzymes: indoleamine 2,3-dioxygenase 1 (IDO1), indoleamine 2,3-dioxygenase 2 (IDO2), and tryptophan 2,3-dioxygenase 2 (TDO2) [73]. Several studies have highlighted the role of Kyn-associated enzymes in cancer [74,75,76]. In CRC, the expression and activity of IDO1 and its effect on tumor-infiltrating lymphocytes (TILs) have already been reported. Interestingly, a significant reduction in TILs was observed in IDO^high^ compared to IDO^low^ expression specimens [77]. Furthermore, Bishnupuri et al. found out that IDO1 and KPM (Kynurenine derived metabolites) cooperate to boost the PI3K-AKT axis to increase cell proliferation, thus contributing to carcinogenesis. The PI3K-AKT axis then promotes the translocation of β-catenin into the nucleus thereby increasing cell proliferation and resistance to apoptosis (Figure 1e) [78]. Of note, the same group has also proposed a link between the oncogenic Myc pathway, tryptophan metabolism, and CRC. They observed that Myc not only increases the level of tryptophan in the tumor cells, but also upregulates the expression of tryptophan solute membrane carriers. In this setting, Kyn has accumulated in the tumor tissue compared to adjacent normal tissue (Figure 1e) [79].

### 2.6. Microbial Metabolites, Immune System, and Host Metabolism: A Tripartite Interplay That Shapes CRC

CRC is a complex pathology that arises from multiple factors ranging from genetic/epigenetics mechanisms to environmental and nutritional mechanisms [80,81]. Recently, the influence of the gut microbiota on intestinal pathophysiology has received a lot of attention. Due to the high number and diversity, the gut microbiota can be viewed as an independent organ or organism in the intestine. In the past, interest has mainly been driven by the characterization of microbial communities, but when the first line of evidence showed the prominent role of the microbiota in several diseases, much effort was made to gain better understanding of the host–microbiota interactions in health and disease [82]. Although more and more publications show the connection between specific microbial communities and CRC, the field is still at its infancy as it is still unclear whether the emergence of some bacteria is the cause or consequence of tumorigenesis. Some microbial signatures have been linked to an increased risk of developing CRC [83]. In addition, perturbations of the microbiota composition (termed dysbiosis) are observed in CRC [84]. Dysbiosis occurs when commensal microbes that usually reside in symbiosis with IECs in their niche in the intestinal environment, are outcompeted by opportunistic microbes that are potentially harmful [85]. Certain microbes such as *Fusobacterium nucleatum* have already been characterized as tumor promoting bacteria [86,87]. However, the modalities through which the microbiota performs its tumor-promoting functions are diverse and not yet fully understood. These microbes can trigger intestinal inflammation, suppress adaptive and innate immunity and lead to the production of oncometabolites, thereby promoting cancer development and metastasis [88].

The intestine itself has been underestimated in terms of metabolic capacity. Additionally, the gut microbiota itself can be viewed as an organ with a massive metabolic performance. Therefore, understanding the metabolic crosstalk between the host and the microbial communities is critical in identifying the mechanisms behind metabolic maladaptation and cancer. The following section provides a comprehensive overview of how microbiota-associated metabolites influence intestinal carcinogenesis and how they can influence the outcome of personalized and conventional antitumor therapies.

### 2.7. Short-Chain Fatty Acids (SCFAs) in CRC: Friends or Foes?

**Figure 2:**
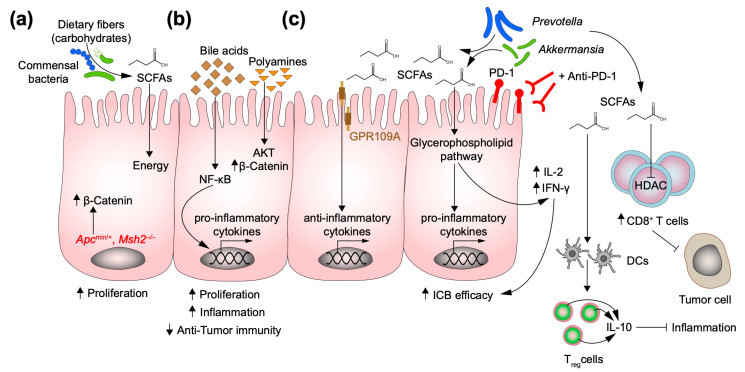
Schematic representation of how non-cancer-cell autonomous (microenvironmental) metabolites affect or protect against the development of CRC: (**a**) Dietary carbohydrates, such as butyrate, lead to hyperproliferation of the intestinal epithelium in a mouse model of intestinal cancer due to increased levels of β-catenin, which translocates into the nucleus and activates genes that promote proliferation. (**b**) Secondary bile acids and polyamines are products of cholesterol and protein metabolism, respectively. Bile acids activate the NF-κB pathway, which leads to induction of pro-inflammatory genes, while polyamines are responsible for activating the PI3K-AKT pathway and thereby increase cell proliferation. (**c**) Short-chain fatty acids (SCFAs) like butyrate can have a tumor protective role. They enter the cell through its receptor GPR109A and promote the expression of anti-inflammatory genes and the priming of dendritic cells (DCs), which leads to differentiation into IL-10 producing Tregs, and thus to the amelioration of colitis. Butyrate also supports the tumor immunity derived from immune checkpoint inhibitors (ICI), which enables the activation of the glycerophospholipid pathway, which in turn upregulates IL-2 and IFN-γ in the tumor microenvironment (TME) and thereby influences the effectiveness of ICB therapy. Outside of IECs, butyrate acts as an HDAC inhibitor in CD8^+^ T cells, maintains the activation of CD8-associated genes, and thus leads to an effective anti-tumor response. NF-κB: Nuclear factor kappa-light-chain-enhancer of activated B-cells; GPR109A: G-protein-coupled receptor 109A; Tregs: T regulatory cells. HDAC: Histone deacetylase. The reported oncometabolites are derived from food and microbial activities.

Besides the ability of the gut microbiome to produce endogenous metabolites, its impressive metabolic capacity is actively involved in the processing of food components such as fibers, lipids, and proteins. Within this variety of micro- and macronutrients, the microbial fermentation of dietary fiber and carbohydrates produces SCFAs. In the previous section, we discussed their roles in intestinal barrier homeostasis and in orchestrating gut-immune homeostasis [30,31]. In addition, several studies examined the role of SCFAs in CRC [89,90]. Three SCFAs have been well known to play key roles in cancer: acetate, propionate, and butyrate. Butyrate is pivotal for colonocytes as it is the main source of energy in hypoxic conditions [91,92]. Acetate and propionate are more frequently found in the circulatory system, so that they are preferentially absorbed by the colonic epithelial cells [93]. Belcheva et al. demonstrated a clear interplay between diet, inflammatory processes, and microbiota, and how this axis promotes CRC. Using a mouse model of intestinal cancer (*Apc*^min/+^), which was additionally deficient for DNA repair (*Msh2*^−/−^), the authors reported that the number of tumor lesions was reduced when animals were treated with antibiotics. Remarkably, the same observation could be made when mice were exposed to a low carbohydrate diet, with no alteration in the microbial community. To investigate the role of SCFAs in tumor development and their metabolism by microbiota, they introduced butyrate through the diet and observed that a diet high in butyrate increased the proliferation of IECs and thus promoted tumorigenesis (Figure 2a) [94]. This study clearly suggests that butyrate is an oncometabolite. However, there is still no consensus as to whether butyrate always acts pro-tumorigenic. Chen et al. examined the role of high-dietary fiber intake in the progression from adenoma to CRC. Screenings of human stool samples classified the SCFAs as tumor-protecting, as higher SCFA yields correlated with a lower risk of developing CRC [95]. Finally, Bultman et al. suggested a role for butyrate in cancer prevention [96]. Since butyrate is not the favorable substrate for energy, cancer cells preferentially use glycolysis (Warburg effect), which results in an accumulation of intracellular butyrate. Moreover, non-metabolized butyrate accumulates in the nucleus of cancer cells, where it acts as a histone deacetylase (HDAC) inhibitor, which prevents the transcriptional activation of tumor promoting transcription factors and induces apoptosis. The discussion of SCFAs and their role in cancer promotion/prevention is still under debate and more and more refined studies will be required to further elucidate the role of this microbially-derived metabolite.

### 2.8. Polyamines and Bile Acids: Oncometabolites Involved in CRC

The metabolism of polyamines is important for both the normal cell growth and the growth of cancer cells. Polyamines have a group charged with a polycation and are involved in several cellular pathways such as protein and nuclei acid synthesis, chromatin rewiring and protection against oxidative stress. In cancer, the metabolism of polyamines is dysregulated and has also been found to interact with oncogenic signaling such as the Wnt pathway. For instance, the enzyme responsible for the polyamine biosynthesis, the ornithine decarboxylases (ODC), is a target of Wnt [97,98]. In addition to the host metabolism of polyamines, the gut microbiota can also biosynthesize polyamines. Some pathogens rely on polyamines for their virulence [99]. Since the energetic demand of cancer cells to sustain their proliferation is higher than that of non-malignant cells, polyamines predominantly accumulate in cancer cells. A potential tumor promoting role of polyamines and bacterial biofilms has consequently been investigated. Johnson et al. demonstrated that CRC patients with bacterial biofilm showed increase in polyamines (N^1^, N^12^-diacetylspermine), which altered the cancer’s metabolism and prepared the way for oncogenic transformation of IECs [100,101]. Furthermore, Goodwin et al. have linked chronic inflammation caused by enterotoxigenic *Bacteroides fragilis* infection to the regulation of spermine oxidase (SMO), the enzyme involved in the spermine metabolism, as a potential axis in promoting colon carcinogenesis [101]. Regulation of intracellular polyamines can also be achieved by controlling polyamine levels. Wang et al. assessed that spermidine/spermine N-acetyltransferase (SSAT), a catabolic enzyme, is a key protein in the metabolism of polyamines. Targeted inhibitions of SSAT increase intracellular polyamines and have a direct effect on the phenotype of CRC cells by promoting cell proliferation, migration, and invasion. Mechanistically, SSAT suppresses the AKT-GSK-3β (glycogen synthase kinase 3β)-β-catenin pathway and thereby inhibits the translocation of β-catenin into the nucleus and stabilizes the expression of EMT-associated genes (Figure 2b) [102].

Bile acids and in particular secondary bile acids are metabolic products of the cholesterol in the liver. The vast majority of secondary bile acids is reabsorbed both from the liver and the intestinal circulation. However, a remaining part in the intestine can be a metabolic target of the gut microbiota, which further process them into unconjugated forms. The most abundant secondary bile acid in humans is deoxycholic acid (DCA) [93]. The first line of evidence that DCA can promote CRC dates back to 1940 [103]. Much data have now accumulated in support of the hypothesis of DCA as an oncometabolite that induces CRC [104]. A prospective study of the microbial composition of the gut in a high vs. low-risk population for developing CRC showed that urine and stool samples from the high-risk population were enriched in bile acids, suggesting a high-fat diet as a nutritional plan [105]. In a recent study with metabolomics analyzes, Yachida et al. demonstrated that the accumulation of bile acids in human adenoma and carcinoma samples coincides with a selective abundance of *Fusobacterium nucleatum*, *Atopobium parvalum,* and *Actinomyces odontolyticus* [106]. DCA also affects the intestinal mucosal barrier and thus promotes intestinal tumor development. Liu et al. showed that administration of DCA to *Apc*^min/+^ mice dramatically increased the number and size of intestinal tumors. Mechanistically, the increased permeability of the intestinal barrier due to DCA administration resulted in activation of the NLRP3 inflammasome and cytokine production, which led to a low-grade inflammation and the formation of a tumor-promoting environment [107]. DCA could also affect the mitochondrial compartment and induce metabolic stress, leading to an increase in ROS (reactive oxygen species) and NF-κB activation. The activated NF-κB then translocates into the nucleus where it activates pro-inflammatory and anti-apoptotic genes (Figure 2b) [108]. Collectively, these results suggest a prominent crosstalk of polyamines, bile acids, and microbiota to induce CRC development.

## 3. Microbial Metabolites Modulate the Immune Microenvironment and the Efficacy of Anti-Tumor Therapies

Cancers consist of a heterogenous population of cell types, such as tumor cells, immune cells, fibroblasts, and endothelial cells, all of which together form the tumor microenvironment (TME) and shape the anti-tumor immunity response [81]. However, the interaction between cancer cells and stroma cells is not the only one that shapes tumor development. Notably, a huge number of studies describe the role of the microbiota in cancer and particularly in CRC, since microbes can physically interact with IECs and develop dynamic crosstalk [109]. In addition, the gut microbiota can not only establish a connection with tumor cells but can also interact with resident stroma and immune cells. This interaction might lead to two opposing outcomes, namely activation or suppression of anti-tumor immunity [110]. Microbial metabolites are one way by which the gut microbiota interacts with tumor cells and the host’s immune environment. In this regard, SCFAs such as butyrate, propionate, and acetate, previously mentioned for their controversial role as oncometabolites, have recently been extensively studied for their therapeutic role in inflammatory and autoimmune diseases [111,112,113]. Besides constituting energy sources for colonocytes, SCFAs shape effector functions of CD4^+^ T cells and impact on differentiation of Tregs. Sing et al. defined the role by which butyrate suppresses inflammation and carcinogenesis in the colon. GPR109A is a G-protein-coupled receptor that can bind butyrate. Mouse models deficient for GPR109A (*Niacr1*^−/−^) showed a significant reduction in colonic Tregs and Treg-mediated IL-10 production and limited the ability of dendritic cells (DCs) and macrophages to induce differentiation of Tregs. Moreover, in GPR109A deficiency in an *Apc*^min/+^ background was found to promote and accelerate colon carcinogenesis (Figure 2c) [114]. Novel reports have found that butyrate, and to a lesser extent propionate, can affect CD8^+^ T cell effector functions. In this regard, Luu et al. showed that butyrate and propionate increased the expression of IFN-γ and granzyme B of CD8^+^ T cells. Mechanistically, butyrate induces histone deacetylation through HDAC inhibition and this leads to an increase in the expression of CD8^+^ T cell effector molecules (Figure 2c) [115]. All these studies highlight the possibility to exploit microbiota-derived metabolism as new therapeutic target for cancer diseases. Since the microbiota influences innate and adaptive tumor immunity, it is reasonable to assume that they may play a role in modulating the anti-tumor immunity that is induced after chemotherapy, radiotherapy, and immunotherapy. It is estimated that roughly 50% of cancer patients undergo radiotherapy with less than half benefiting from it. Although the concept of radiobiology has not received so much attention recently due to the advent of next generation anti-tumor treatments like chemotherapy and immunotherapy, improving the quality of life and minimizing the side effects of patients who receive this treatment is still a major concern [116]. Radiation-induced injuries and in particular radiation-induced intestinal injuries have raised a lot of interest due to the growing body of evidence linking gut microbiota and effectiveness of radiotherapy. However, there is still no consensus in the field on the interplay between microbiota and radiation therapy [117]. One of the main consequences after radiotherapy is the appearance of a microbial dysbiosis. Several studies have already shown the effect of radiotherapy on the abundance and diversity of the gut microbiome [118,119]. Conversely, the microbiota is able to influence the anti-tumor immune response in radiotherapy. Interestingly, Yang et al. reported that microbiota-derived butyrate impairs IFN-γ-mediated anti-tumor immunity after ionizing radiation. The accumulation of this metabolite in tumor tissue restrained CD8^+^ T cell activation through impairment of the antigen presentation machinery of DCs [120]. Another study claimed that the tryptophan and propionate metabolism have radioprotective properties. Guo et al. analyzed the microbiota composition of mice recovering from radiation exposure. In the “survivors” group the authors noticed a rearrangement of the taxonomic representation, with *Lachnospiraceae* and *Enterococcaceae* being significantly enriched [121]. Moreover, Valeric acid (VA), another metabolite derived from gut commensals, was investigated for its effects on radioprotection. VA is a SCFA, which is obtained from the fermentation of insoluble fiber from the gut microbiota. Li et al. reported that VA helps to mitigate radiation-induced effects in the intestine by restoring the integrity of the intestinal epithelia barrier through overexpression of Keratin 1 [122].

Unleashing anti-tumor T cell responses that block inhibitory T cell signaling pathways has revolutionized the era of cancer treatment. Immune checkpoint inhibitors (ICI) are monoclonal antibodies that target specific membrane receptors on the surface of T cells, thereby blocking any inhibition by tumor cells. Cancer patients have benefited a lot from immunotherapy with a better overall survival. However, only a small fraction of patients responds well to ICI [123,124]. The reasons for this are still not completely understood. It is worth noting that emerging studies are highlighting the role of microbiota in shaping the immune responses upon ICI treatment [125]. In this regard, CRC shows a paucity of “responder” patients. This fraction is constituted by patients that harbor mutations in DNA mismatch repair machinery (pMMR, alternatively called microsatellite instability, MSI). Xu et al. suggested that the effectiveness of anti-PD-1 treatment in microsatellite stable (MSS) subtype of CRC is shaped by the microbiota via the glycerophospholipid pathway. They noticed that the tumor grow was dampened in tumor mice treated with anti-PD-1 without antibiotics, while the administration of antibiotics upon PD-1 treatment did not result in tumor shrinking. Metabolomics analyses revealed that one of the most influenced pathways was the glycerophospholipid pathway (Figure 2c) [126]. Analogous results were obtained by Vetizou and colleagues who compared the therapeutic efficacy of anti-CTLA4 against sarcomas in mice under both specific-pathogen-free (SPF) and germ-free (GF) conditions. Tumor growth was reduced in SPF compared to GF mice. Moreover, they identified specific species of bacteria (*B. fragilis*, *B. thetaiotaomicron,* and *Burkholderiales*), which activated Th1 responses against the tumor [127]. However, some metabolites might have a negative impact on ICI. A recent study highlighted the role of some metabolites to be potentially considered as biomarkers of responsiveness to ICI [128]. Botticelli et al. screened the fecal microbiota of 11 non-small cell lung cancer (NSCLC) patients that received anti-PD-1 treatment. Two categories were observed: early responders (ER) and long responders (LR). Further metabolomics analysis revealed the association of responsiveness with the accumulation of specific metabolites. LR were characterized by high levels of SCFAs, while ER showed increases in p-Cresol and tridecane, which had previously been associated with toxicity and intestinal barrier dysfunction [129,130]. Overall, these data underline the role of the microbiota as a mediator in relation to the therapeutic efficacy of anti-tumor therapy and the possibility of using microbiota-based remedies as biomarkers for efficacy.

## 4. Conclusions

The aim of this review was to elucidate the role of metabolites as novel molecular messengers in intestinal diseases. Since some intestinal metabolites exert similar functions, such as maintaining homeostasis or fostering metabolic diseases (e.g., due to their intracellular accumulation), the study of metabolites as messengers between health and disease has changed our view of intestinal homeostasis and pathological states. Although we have made rapid advances in characterizing the intrinsic cellular metabolism, the depth of metabolomics approaches is not yet sufficient to comprehensively describe the regulation/alteration of metabolic pathways and their contribution to the communication between host cells and the environment. In light of recent findings involving microbiota-derived metabolites and their role in health and disease, a collective effort is necessary to understand the relationship between environmental metabolites (diet- and microbiota-derived metabolites), host-derived metabolites and the intestinal epithelium. Rewiring the cellular metabolism through intrinsic or extrinsic factors helps to adapt to environmental stimuli, and thus any maladjustment can lead to a changed metabolism and to disease development. In this context, the intestine has been recognized as a metabolic organ in itself that can produce many different metabolites that not only act locally (i.e., towards other IECs) but also systemically (inter-organ communication such as gut–brain or gut–liver axis). IECs are a central hub to segregate microbial stimuli and signals from the immune cells residing in the lamina propria. Defects in this miscommunication lead to intestinal disorders like IBD and CRC. Accumulation of some oncometabolites like 2-DHG, kynurenine, and bile acids have been demonstrated to promote tumorigenesis in the colon (Table 1). This dysfunctional accumulation of oncometabolites is driven by oncogenic signaling within cancer cells or by cell extrinsic stimuli derived from the microbiota. The pioneering discoveries in the field of microbiota have paved the way for the newly identified mechanisms of the IEC–microbiota-crosstalk. The gut microbiota can be considered as another organ in the gastrointestinal tract that has its own regulatory mechanisms. An unbalanced distribution of commensal bacteria opens the way for disease-associated microorganisms. This condition, known as dysbiosis, is often linked to the risk of developing bowel inflammation and colon cancer. Seminal studies in mice uncovered several mechanisms by which microbiota-derived metabolites might promote or block tumor progression. Thus, the translation of these findings to human patients has motivated the assumption that there are microbial components (metabolites) that are essential for strengthening anti-tumor immunity. Finally, recent evidence has highlighted the protective role of microbial species during radiotherapy and the ability to shape immunotherapy. Hence, in the future, more research focusing on the crosstalk between the intrinsic metabolism of IECs and the microenvironmental metabolites will provide more insights into disease development and thus unveil new therapeutic vulnerabilities.

## Figures and Tables

**Table 1:** Evidence of known food and microbial-derived metabolites in CRC: α-KG: alpha ketoglutarate; CSC: cancer stem cells; D2HGDH: D-2-hydroxyglutarate dehydrogenase; EMT: epithelial-mesenchymal transition; HDAC: Histone deacetylase; LAT1: L-type amino acid transporter; NLRP3: NLR family pyrin domain containing 3; SCFAs: Short-chain fatty acids; Tregs: T regulatory cells.

Metabolite	Origin	Mechanism	Effect	Reference
SCFAs (butyrate, propionate, acetate)	Food	Treg-mediated immunity	Gut-immune regulation	
	Microbial fermentation products	Inhibition of HDACs	Increased intestinal barrier integrity	[29,30,31]
		Tumor cells apoptosis	Tumor protective	
Butyrate	Food	Proliferation	Proliferation in *Apc*^min/+^, *Msh2*^−/−^ mice	[94,95]
	Microbial fermentation products	Enhanced *β*-catenin activity	Tumor promoting	
Vitamin A/D	Food	Reduction in cytokines levels	Increased intestinal barrier integrity	[33,34,35]
		Upregulation of Tight junctions	Tumor protective	
		Increased intestinal barrier integrity		
Polyamines	Food, microbial contents	Upregulation of spermine oxidase	Inflammation	
		Upregulation of EMT genes	Proliferation	[99,101,102]
			Tumor promoting	
Deoxycholic acid (DCA)	Cholesterol/secondary bile acids	Pro-inflammatory genes/anti-apoptotic genes	Intestinal barrier dysfunction	
		NLRP3 activation	Cytokine inflammation	[107,108]
		Intestinal barrier permeability	CSC proliferation	
			Tumor promoting	
2-hydroxyglutarate (2-HG)	Reduced form of *α*-KG	Promotion of EMT genes	Invasiveness and metastasis	
		Downregulation of D2HGDH	EMT phenotype	[69,71,72]
			Apoptosis resistance	
			Tumor promoting	
Kynurenine	Diet-derived tryptophan	PI3K-AKT-induced proliferation	Proliferation	
		Upregulation of membrane carrier LAT1	Biomass, protein synthesis	[78,79]
			Tumor promoting	
